# “Rejection Makes Me Suspicious”: Complex Temporal Network Approach to the Dynamics of Real-Time Paranoid Thoughts and Psychological Vulnerability

**DOI:** 10.1093/schizbullopen/sgaf021

**Published:** 2025-09-25

**Authors:** Paulina Bagrowska, Łukasz Gawęda

**Keywords:** paranoia, paranoid thoughts, vulnerability, ESM

## Abstract

**Background and Hypothesis:**

Theoretical models and empirical evidence suggest that paranoid thoughts stem from a heightened sense of vulnerability, including the perception of the world as dangerous, and fears of rejection and social evaluation. However, the factors contributing to this sense of vulnerability and the complex interplay between these elements remain underexplored.

**Study Design:**

A total of 175 individuals recruited from a nonclinical community sample, including 103 participants with low levels of paranoid thoughts (LP) and 72 with high levels (HP), took part in a 7-day ESM study assessing momentary levels of paranoia, social functioning, social rejection, negative affect, body image, and misophonia symptoms. Temporal, contemporaneous, and between-subject network models were estimated for the total sample and its subgroups separately.

**Study Results:**

The temporal network identified paranoid thoughts as a predictor of negative affect, feelings of rejection, and negative body image, while in turn being predicted by feelings of rejection and reduced social safety. A bidirectional relationship between paranoia and social rejection emerged. These findings were supported by contemporaneous and between-subject networks, which showed that paranoid thoughts co-occurred with and were, on average, linked to feelings of rejection, negative affect, and reduced social safety.

**Conclusions:**

These findings suggest that social rejection plays a central role in paranoia. While group differences in network structure were modest, the HP group exhibited more numerous and stronger connections between variables, suggesting that paranoia may develop through the gradual reinforcement of connections between symptoms rather than structural shifts, highlighting the importance of early intervention.

## Introduction

Paranoid thoughts, observed in clinical and general populations,^1^ refer to excessive mistrust and unfounded beliefs in hostile intentions from others.^2^^,^^3^ While these thoughts typically resolve naturally over time, the interaction of various factors can lead to their progression into full-blown disorders.^4–6^ Research suggests that paranoia builds on feelings of vulnerability—concerns about social evaluation, fears of rejection, and the perception of the world as a dangerous place. These fears are conceptualized as interpersonal sensitivity^7^—“feeling vulnerable in the presence of others due to expectation of criticism or rejection.”^8^ A recent study^9^ suggested that reducing the sense of vulnerability may be one of the key pathways to lowering paranoia. Therefore, exploring the foundational element of the paranoia hierarchy^1^—interpersonal sensitivity—and identifying its potential predictors and interactions between them could be crucial for designing effective interventions.

Feelings of vulnerability include fears of social rejection.^1^^,^^9^ Numerous studies have shown that social rejection can trigger paranoia, often through an increase in negative emotions.^10–13^ Past adverse experiences, such as actual or perceived social rejection, can significantly influence how individuals perceive social cues and engage in human interactions.^14^ These experiences may increase rejection sensitivity—an anxious anticipation and defensive overreaction to stimuli or situations that may involve rejection,^15^ which may further result in hypervigilance in social contexts, causing individuals to misinterpret others’ intentions as hostile despite insufficient evidence.^16^ On the other hand, there are negative self-beliefs and fears of social evaluation.^1^^,^^9^ Previous research has extensively examined the relationship between self-esteem and paranoid thoughts,^17–21^ indicating that low and unstable self-esteem significantly predicts both trait and state paranoia. Additionally, emerging research links paranoid thoughts to negative body image—one aspect of self-esteem.^22–26^ While the association between self-esteem and paranoia is well established, body image remains a newer research focus, with its causal role largely unexplored. Importantly, interpersonal (or rejection) sensitivity has been suggested to mediate the relationship between negative self-views and paranoia,^24^^,^^27^ but the interplay between these factors is yet to be examined, especially in the light of another aspect of vulnerability—the perception of the world as dangerous and the sense of loss of control over events.^1^^,^^9^ For instance, recent studies have linked paranoid thoughts to misophonia symptoms,^28^ a form of decreased sound tolerance, particularly to sounds made by other people, which places it strongly within an interpersonal context.^29^ Exposure to misophonia trigger sounds has been shown to induce negative emotions, further leading to the intensification of paranoid thoughts.^30^ Since triggers like chewing, swallowing, and breathing are often unavoidable—making the world feel unpredictable, uncontrollable, and thus dangerous—individuals with misophonia may experience constant stress, anxiety, and feelings of inferiority (e.g., “I am a bad person because of my reactions”), further heightening their sense of vulnerability to harm, which serves as the foundation for paranoia development. Although these factors have been studied individually in the past, there is a lack of research that examines multiple “vulnerability factors” in the context of paranoia together. A recent network approach helps to address this gap by enabling the simultaneous study of multiple factors and the development of sophisticated models that capture their dynamics.

The network approach to psychopathology^31^ posits that mental disorders develop from causal interactions between multiple symptoms in a network.^32^ Recent studies have employed this approach to paranoia research,^33–36^ but the findings are based on cross-sectional data, employing a rather static approach. Thus, directional relationships between factors and their temporal dynamics cannot be established. To the best of our knowledge, only one study to date has applied a temporal network approach^37^ to investigate paranoia.^38^ However, this was a pilot study conducted on a relatively small sample, indicating a need for further research.

Hence, this study employs a temporal network approach and an ecologically valid, intensive longitudinal experience sampling method (ESM) to identify key predictors and outcomes that reflect broader psychological mechanisms, such as social evaluative concerns, fears of social rejection, and perceived interpersonal threat, within a network of vulnerability-related factors in the context of paranoid thoughts and to explore their dynamic temporal interplay.

## Materials and Methods

This study forms part of a larger project on the experimental testing of the role of interpersonal sensitivity and fear conditioning in the context of paranoia.

### Participants

One hundred and seventy-five individuals (58.3% of females) were recruited from the nonclinical community sample in two ways—via a survey link shared on social media and via a survey panel using the CAWI (computer-assisted web interview) method. The study comprised a pilot, an initial screening cohort of over 800 individuals to establish cutoff scores for the level of paranoid thoughts, using the Revised Green et al. Paranoid Thoughts Scale (R-GPTS),^39^ based on which participants were subsequently being recruited and assigned to one of two groups. Namely, the control group (with low levels of paranoid thoughts; LP) consisted of participants scoring within the lowest 10% on the R-GPTS scale, as determined during the initial screening phase and corresponding to a score of ≤1 point. In contrast, the experimental group (with high levels of paranoid thoughts; HP) consisted of individuals scoring within the highest 10%^40–42^ on the R-GPTS scale, corresponding to a score of ≥35 points. The study included a total of 103 participants in the LP group (52.4% of females) and 72 participants in the HP group (66.7% of females).

The study focused on adults aged 18-40. Subjects assigned to the LP group and included in the study were required to score ≤1 point on the R-GPTS scale, have no active symptoms of any psychiatric disorders in the past month, and not have used any psychotropic medication in the past month. HP participants were included in the study if they scored ≥35 points on the R-GPTS scale. Individuals from both groups were excluded from the study if they were outside the age range of 18-40, had a history of neurological disorders or intellectual disability, had been diagnosed with autism spectrum disorders, had a lifetime history of psychotic disorders or bipolar disorders (as the study aimed to examine nonclinical paranoid thoughts in the general population), reported a history of psychotic disorders or bipolar disorders in their first-degree relatives, had ever used antipsychotic medication, or had declared alcohol or substance abuse or dependence in the past 6 months. Given the co-occurrence of elevated paranoid thoughts with other symptoms, including anxiety and depression, the presence of concomitant mental disorders was not an exclusion criterion in the HP group. The study was approved by the Ethics Committee of the Institute of Psychology of the Polish Academy of Sciences (no. 03/III/2021) and conducted in accordance with the latest version of the Declaration of Helsinki. All the participants were required to provide written consent at each stage of the recruitment process and the main study.

### Measures

The specific measurements and items used in the ESM assessment are detailed in Table 1. The factors measured in the ESM include paranoid thoughts, social functioning (social isolation, social stress, social safety), feeling of social rejection, important/stressful events, negative affect, negative body image and misophonia symptoms.

**Table 1:** ESM Measures

**Domain/Factor**		**ESM measures/items**
Paranoia		The total “paranoia” score was calculated as a mean of 6 items. The Likert scale was employed, with responses ranging from “1 – Definitely not” to “7 – Definitely yes”. The items included: “Right now, I am distrustful”, “Right now, I think that others may want to intentionally hurt me”, “Right now, I think others are conspiring against me”, “Right now, I think bad things are being said about me behind my back”, “Right now, I think I might be being watched or followed”, “Right now, I think that people are deliberately being hostile towards me”. Higher scores indicated a higher level of paranoia-like thoughts (between-subject Cronbach’s *α* = 0.93).
	Social isolation	The “social isolation” score was calculated based on a question “Who are you with right now?”. The respondents were asked to select one or more of the following options: “alone”, “with family”, “with a partner”, “with friends”, “with strangers”, or “with coworkers”. Those who indicated that they were “alone” were assigned a value of “1”, while all other responses were assigned a value of “2”, indicating that the participants were in the company of someone else.
Social functioning	Social stress	The “social stress” score was calculated based on the “social isolation” score. In the event that a participant indicated that they were currently alone, they were requested to indicate on a 1-7 Likert scale, with responses ranging from “1 – Definitely not” to “7 – Definitely yes”, whether they “... would prefer to have a company right now”. In the event that a participant indicated that they were currently in the company of others, they were asked to indicate whether they “... would prefer to be alone right now”. Higher scores indicated a higher level of social stress.
	Social safety	The “social safety” score was calculated based on the “social isolation” score. In the event that participants indicated that they were with other people right now, they were prompted to provide responses to two statements: “Right now, I feel accepted by the people I am currently with”, and “Right now, I feel threatened by the people I am currently with”. The responses ranged from “1 – Definitely not” to “7 – Definitely yes”. Higher scores indicated a higher level of social safety [feeling threatened was reverse coded].
Social rejection		The feeling of being rejected by others was assessed with one item, that is, “To what extent do you feel rejected or overlooked by other people right now?”. The Likert scale was employed, with responses ranging from “1 – Definitely not” to “7 – Definitely yes”. Higher scores indicated a higher level of feeling rejected by others.
Event		The “important event” (assessing minor daily stress) score was calculated based on one question: “Think of the most important event that has happened since the last 'beep'. Rate how pleasant or unpleasant the event was”. The Likert scale was employed, with responses ranging from “1 – Very unpleasant” to “7 – Very pleasant”. Higher scores indicated more pleasant event.
Negative affect		The total “negative affect” score was calculated as the mean of five items. The Likert scale was employed, with responses ranging from “1 – Definitely not” to “7 – Definitely yes”. Participants were asked to report to what extent they felt a given emotion at that moment. The items covered being “sad”, “worried”, “angry”, “ashamed”, and “irritated”. Higher scores indicated a higher level of negative affect (between-subject Cronbach’s *α* = 0.9).
Body image		Perceived body image was assessed with one item, that is, “What feelings do you have about the appearance of your body right now?”. The Likert scale was employed, with responses ranging from “1 – Very unpleasant” to “7 – Very pleasant”. Higher scores indicated more positive body image.
Misophonia		Misophonia symptoms were assessed with one item, that is, “Do you feel unpleasant emotions because of sounds made by other people (eg, smacking, chewing, sniffling, breathing, or others) right now?”. The Likert scale was employed, with responses ranging from “1 – Definitely not” to “7 – Definitely yes”. Higher scores indicated more misophonia symptoms.

The R-GPTS^39^ was employed to measure the baseline level of paranoid thoughts experienced over the past month. It consists of 18 items forming two subscales: one to assess ideas of reference (8 items) and another to assess ideas of persecution (10 items). The responses ranged from 0 to 4, resulting in a total score between 0 and 72 points. Cronbach’s alpha (*α*) for this scale in this study was 0.98.

The *Comprehensive Assessment of At-Risk Mental States* (CAARMS)^43^ is a semistructured clinical interview designed to assess attenuated psychotic symptoms and to identify individuals at risk for developing psychotic disorders. The CAARMS is structured into 7 domains, including the assessment of positive symptoms, cognitive change, emotional disturbance, negative symptoms, behavioral change, motor changes, and general psychopathology. In the present study, a shortened version of the interview was employed, with only positive and negative symptoms being assessed. Each of the four subscales of positive symptoms (unusual thought content, nonbizarre ideas, perceptual abnormalities, and disorganized speech) and three subscales of negative symptoms (alogia, avolition/apathy, and anhedonia) were rated in terms of the intensity of symptoms (Global Rating Scale) and frequency and duration of the symptoms. Both scales rate symptom severity and frequency on a scale from 0 to 6 for each of the individual positive and negative symptom subscales. This yields a total score for positive symptom intensity ranging from 0 to 24 points, a total score for positive symptom frequency and duration ranging from 0 to 24, a total score for negative symptom intensity ranging from 0 to 18, and a total score for negative symptom frequency and duration ranging from 0 to 18. All the participants were asked to respond to questions pertaining to the experiences occurring in *the past year* and *the past month*.

The *Mini International Neuropsychiatric Interview* (MINI)^44^ is a structured diagnostic interview aiming to assess a range of psychiatric disorders using the established *Diagnostic and Statistical Manual of Mental Disorders* (*DSM-IV*) and *International Classification of Diseases* (*ICD-10*) criteria. Each module corresponds to a distinct disorder and receives a score of 1 if the participant fulfills the diagnostic criteria for that particular disorder or a score of 0 if the diagnostic criteria are not met. In this study, only a subset of disorders were evaluated, including major depressive disorder, suicidality, manic episode, hypomanic episode, bipolar disorder, social anxiety disorders (social phobia), alcohol use disorder, substance use disorder, anorexia nervosa, bulimia nervosa, and body dysmorphic disorder.

The *Polish Adult Reading Test* (PART)^45^ was employed to assess the level of premorbid intelligence. PART is a validation of the National Adult Reading Test (NART) by Hazel Nelson,^46^ which is one of the most widely used methods of measuring premorbid intelligence in psychiatric patients. The PART is comprised of 50 words. Participants are given 1 point for each correctly pronounced word, while incorrectly pronounced words yield 0 points.

### Procedure

To participate in the study, all the individuals were required to complete a preliminary screening questionnaire. This survey was distributed by an external company to research panel participants and shared by the researchers across various social media platforms throughout the study. This questionnaire covered the R-GPTS scale, demographic data, personal and family psychiatric history (including lifetime diagnoses and current symptoms), neurological disorders or intellectual disabilities, history of psychotropic medication use (ever and currently), and psychoactive substance use, including alcohol. Those not meeting the inclusion criteria were thanked for their participation and not invited to take part in further recruitment stages. Individuals who passed the preliminary screening were subsequently contacted for a telephone verification to reconfirm their eligibility, with particular focus on the level of paranoid thoughts within the predetermined cutoff points and revisiting the inclusion and exclusion criteria. If a participant did not qualify at this stage, they were thanked and excluded from further participation. Those who successfully passed both the online screening and telephone verification were invited to the main study phase conducted on-site at the Institute of Psychology of the Polish Academy of Sciences in Warsaw.

The procedure began with the MINI interview to identify the presence of any current and lifetime psychiatric disorders. In the event that a participant met the diagnostic criteria for bipolar disorder or was currently experiencing substance abuse or dependence, the study was terminated for that individual, with no progression to the ESM assessment. Subsequently, participants proceeded with the PART test, which required them to read aloud the words provided to them on a paper sheet. Following this, participants were asked to complete a series of self-report questionnaires, including the R-GPTS. The CAARMS interviews were scheduled in a separate meeting after the ESM phase to avoid overburdening participants with an extensive assessment in a single session and to comply with the demands of subsequent experimental procedures not covered in this study.

The ESM assessment, conducted using the movisensXS GmbH software, began the following day. Participants were asked to complete surveys 8 times a day for 7 consecutive days, resulting in a total of 56 assessments. The surveys were distributed at random times between 9 am and 10 pm, with at least a 45-minute break between each prompt. Participants were allowed to delay completing the survey by up to 11 minutes. If a survey was not answered immediately or within the specified period, it was recorded as not responded. Participants were required to respond to at least 6 out of the 8 daily surveys. To ensure compatibility with the movisensXS app, all the participants were provided with the same model of smartphone. Finally, participants who completed this stage of the study, as well as the subsequent experimental phase (not reported here), received financial compensation in the form of a prepaid card with an approximate value of EUR 115.

### Statistical Analysis

Statistical analyses were performed using SPSS 29 and the R software (R version 4.3.0, RStudio version 2023.03.1+446).^47^

All the analyses were conducted on the total sample, as well as on the LP and HP groups separately for group comparisons. Independent samples *t*-tests were employed to examine the statistical differences between the groups in terms of their demographic characteristics and all the studied variables. The effect sizes were calculated using Cohen’s *d*.

### Network Estimation

Temporal network analysis, aimed at investigating the dynamic interrelationships between paranoia-like thoughts and their correlates, was conducted using the R package multivariate vector autoregression (mlVAR).^48^ The standard vector autoregression (VAR) model^49^ estimates the extent to which one variable at a specific time point (*t*) can be predicted by other variables at a previous time point (*t* − 1). In this study, time-series (ESM) data with a multilevel structure derived from multiple subjects were analyzed. Therefore, the VAR model within a multilevel modelling framework (mlVAR) was implemented, as it allows the temporal dynamics to be studied not only within a single individual but also at the group level, estimating both average and individual effects. Network models were estimated for the total sample, as well as for the LP and HP groups separately.

To address the issue of missing data and to avoid the unnecessary exclusion of valid data, the Kalman filter^50^ for data imputation was employed, using the R package *imputeTS.*^51^ The mlVAR analysis assumes stationarity of all variables, which means that the statistical properties of the time series, such as means, variances, and autocorrelations, should be stable over time.^52^^,^^53^ To test this assumption, the augmented Dickey-Fuller (ADF) test was employed,^54^ using the *adf.test* function within the R package tseries*.*^55^ The ADF tests revealed that all the variables met the assumption of stationarity (all *p*-values < .01).

The mlVAR analysis on time-series data allows for estimation of three networks: temporal, contemporaneous, and between-subject networks. All three network models were visualized using the R package *qgraph.*^56^ In all cases, each node (depicted as a circle) represents a variable. The variables in the networks include paranoid thoughts (“Paranoia”), negative affect (“NegAffect”), feeling of social rejection (“FeelReject”), important event (“Event”), social stress (“SStres”), social safety (“SSafety”), body image (“BodyImage”), and misophonia symptoms (“Misophonia”). The *temporal network* represents (1) cross-lagged effects, that is, how one variable (measured at *t* − 1) influences another variable (measured at *t*) over time, controlling for the autoregressive effects, and (2) autoregressive effects, that is, how one variable (*t* − 1) is predictive of itself over time (*t*), controlling for the cross-lagged relations.^57^ When one variable (*t* − 1) significantly predicts another variable in the next measurement window (*t*), the nodes are linked with a directed arrowhead line pointing from one node to another (cross-lagged effects). An arrow of a node pointing at itself represents an autoregressive effect. The *contemporaneous network* represents partial correlations between nodes measured at the same time point, after controlling for both temporal effects and all other variables in the network in the same window of measurement.^37^ Significant partial correlations between nodes are depicted as lines without arrowheads. The *between-subject network* represents the average relationships between variables across individuals.^58^ It demonstrates how, on average, the level of one variable is related to the level of another variable across multiple participants over time (ie, the entire ESM testing period) while accounting for the influence of other variables in the network. In all cases, thicker lines indicate stronger effects, green lines indicate positive effects, and red lines indicate negative effects. The absence of a line between two nodes indicates no statistically significant associations.

In temporal networks, two commonly calculated centrality indices are in-strength and out-strength.^52^ Out-strength indicates the summed absolute strengths of all outgoing edges, representing the extent to which a specific node predicts other nodes. In-strength, on the other hand, indicates the summed absolute strengths of all incoming edges, representing the extent to which the node is predicted by other nodes in the network. Given that our network contains negative edges, we calculated the in- and out-expected influence indices, rather than using the in- and out-strengths. Expected influence operates in a manner analogous to strength, but it takes into account the directional nature of the edges between nodes; that is, it is not dependent on the absolute values of the edge weights. Nevertheless, despite the calculation of centrality indices, it has been advised that the centrality measures in temporal networks should be interpreted with particular caution.^59^

### Comparison of Group Networks

In order to estimate group differences in all three types of networks (LP vs HP), permutation tests with 1000 permutations were performed, using the R-package *mnet.*^60^^,^^61^

## Results

### General Sample Characteristics

The total sample characteristics are presented in Table 2, while Table 3 provides a breakdown of the demographic characteristics for the two subgroups (LP and HP). No significant differences were found between the groups in terms of gender, educational background, or premorbid IQ. Nevertheless, significant differences were observed in age, with LP participants being slightly older. The HP group reported a higher prevalence of psychiatric diagnoses and psychotropic medication use, as well as a greater number of diagnostic criteria met for various psychiatric disorders based on the MINI interview. Those in the HP group displayed heightened levels of baseline paranoia-like thoughts, as well as both positive and negative attenuated psychotic symptoms, as assessed by the CAARMS interview. Moreover, the groups differed significantly across nearly all the ESM variables, with the exception of “social isolation”. In the LP group, men reported higher levels of feelings of rejection, social stress, and lower social safety compared to women. Within the HP group, men exhibited significantly higher levels of paranoid thoughts than women. The mean completion rate for all ESM surveys was 95% (SD = 0.06; range 70%-100%).

**Table 2:** Descriptive Characteristics of the Total Sample (*n* = 175)

	***n* (%)**		***M* (SD)**	**Range**
Gender		Age	29.16 (6.79)	18-40
Female	102 (58.3)	PART (premorbid IQ)	48.04 (2.73)	29-50
Male	73 (41.7)	R-GPTS	14.27 (18.97)	0-70
Education		Reference	7.75 (9.68)	0-32
Primary	0 (0)	Persecution	6.51 (9.69)	0-40
Vocational	2 (1.1)	CAARMS positive (past year)		
Secondary	81 (46.3)	Score	2.66 (3.28)	0-15
Higher	92 (52.6)	Frequency	2.93 (3.18)	0-19
Professional situation		CAARMS positive (past month)		
Employed	135 (77.1)	Score	2.25 (3.05)	0-14
Unemployed	5 (2.9)	Frequency	2.63 (3.58)	0-19
Retired	2 (1.1)	CAARMS negative (past year)		
Student	59 (33.7)	Score	1.56 (2.02)	0-10
Psychiatric disorders (lifetime)	35 (20.0)	Frequency	2.21 (2.99)	0-14
Medication use (lifetime)	52 (29.7)	CAARMS negative (past month)		
Medication use currently	18 (10.3)	Score	1.33 (2.04)	0-10
MINI		Frequency	1.93 (2.92)	0-13
MDD	54 (30.9)	ESM		
Suicidality	20 (11.4)	Paranoia	1.56 (1.1)	1-7
Social anxiety	9 (5.1)	Social isolation	1.60 (0.5)	1-2
AUD	8 (4.6)	% time alone	38.8	
SUD	3 (1.7)	% time with others	57.1	
Bulimia nervosa	6 (3.4)	Social stress	2.64 (1.9)	1-7
BDD	11 (6.3)	Social safety	6.19 (1.1)	1-7
		Social rejection	1.74 (1.4)	1-7
		Event	4.70 (1.6)	1-7
		Negative affect	2.01 (1.4)	1-7
		Body image	4.70 (1.6)	1-7
		Misophonia symptoms	1.53 (1.2)	1-7

Abbreviations: MINI, the Mini International Neuropsychiatric Interview (criteria met for a given diagnosis at any point throughout the participant’s lifetime); MDD, major depressive disorder; AUD, alcohol use disorder; SUD, substance use disorder; BDD, body dysmorphic disorder; PART, Polish Adult Reading Test (premorbid IQ); R-GPTS, the Revised Green et al. Paranoid Thoughts Scale; CAARMS, the Comprehensive Assessment of At-Risk Mental States; ESM, experience sampling method.

**Table 3:** Descriptive Characteristics of the Subgroups

**Measures**	**HP (*n* = 72)**	**LP (*n* = 103)**	**Group comparison**
	***n*** **(%)**		
Gender (females), *n* (%)	48 (66.7)	54 (52.4)	n.s.
Education (higher)	33 (45.8)	59 (57.3)	n.s.
Professional situation (employed)	49 (68.1)	86 (83.5)	*p* < .05; *χ*^2^ = 4.3
Psychiatric disorders (lifetime diagnosis)	25 (34.7)	10 (9.7)	*p* < .001, *d* = 0.7
Medication use (lifetime use)	37 (51.4)	15 (14.6)	*p*< .001, *d* = 0.9
Medication use currently (current use)	18 (25.0)	0	*p*<.001, *d* = 1.1
MINI (diagnostic criteria met)			
MDD	42 (58.3)	12 (11.7)	*p* < .01, *d* = -0.4
Suicidality	18 (25.0)	2 (1.9)	*p*< .001, *d* = −0.8
Social anxiety	9 (12.5)	0	*p*< .001, *d* = −0.6
AUD	5 (6.9)	3 (2.9)	n.s.
SUD	12 (16.7)	17 (16.5)	n.s.
Bulimia nervosa	6 (8.3)	0	*p*< .01, *d* = −0.5
BDD	10 (13.9)	1 (1.0)	*p* < .01, *d* = −0.5
	**M (SD)**		
Age	27.4 (6.8)	30.4 (6.5)	*p* < .01, *d* = 0.5
PART (premorbid IQ)	48.2 (2.1)	47.9 (3.1)	n.s.
R-GPTS	33.0 (16.4)	1.2 (2.4)	*p* < .001, *d* = −3.0
Reference	17.6 (7.6)	0.9 (1.6)	*p*< .001, *d* = -3.3
Persecution	15.4 (9.6)	0.3 (1.1)	*p*< .001, *d* = −2.4
CAARMS positive (past year)			
Score	5.5 (3.1)	0.8 (1.3)	*p* < .001, *d* = −2.1
Frequency	5.8 (3.6)	0.9 (1.7)	*p* < .001, *d* = −1.5
CAARMS positive (past month)			
Score	4.8 (3.2)	0.5 (1.0)	*p* < .001, *d* = −2.0
Frequency	5.6 (3.8)	0.6 (1.4)	*p* < .001, *d* = −1.9
CAARMS negative (past year)			
Score	3.1 (2.3)	0.5 (0.8)	*p* < .001, *d* = −1.6
Frequency	4.2 (3.3)	0.8 (1.8)	*p* < .001, *d* = −1.4
CAARMS negative (past month)			
Score	3.0 (2.3)	0.2 (0.5)	*p* < .001, *d* = −1.8
Frequency	4.2 (3.3)	0.4 (0.9)	*p*< .001, *d* = −1.7
ESM			
Paranoia	2.26 (1.4)	1.08 (0.3)	*p* < .05, *d* = −1.2
Social isolation	1.60 (0.5)	1.61 (0.5)	n.s.
% time alone	39.1	38.6	n.s.
% time with others	57.0	57.1	n.s.
Social stress	3.04 (2.0)	2.37 (1.7)	*p* < .05, *d* = −0.4
Social safety	5.70 (1.2)	6.54 (0.7)	*p*< .05, *d* = 0.8
Social rejection	2.44 (1.7)	1.24 (0.8)	*p* < .05, *d* = −0.9
Event	4.30 (1.7)	4.98 (1.4)	*p* < .05, *d* = 0.4
Negative affect	2.86 (1.6)	1.41 (0.8)	*p* < .05, *d* = −1.2
Body image	3.93 (1.5)	5.24 (1.4)	*p* < .05, *d* = 0.9
Misophonia symptoms	1.97 (1.5)	1.22 (0.8)	*p* < .05, *d* = −0.6

Abbreviations: MINI, the Mini International Neuropsychiatric Interview (criteria met for a given diagnosis at any point throughout the participant’s lifetime); MDD, major depressive disorder; AUD, alcohol use disorder; SUD, substance use disorder; BDD, body dysmorphic disorder; PART, Polish Adult Reading Test (premorbid IQ); R-GPTS, the Revised Green et al. Paranoid Thoughts Scale; CAARMS, the Comprehensive Assessment of At-Risk Mental States; ESM, experience sampling method.

### Temporal Network Estimation

The temporal network models consist of 8 nodes (variables) and a total of 64 edges, including 8 autocorrelation edges.

**Figure 1:**
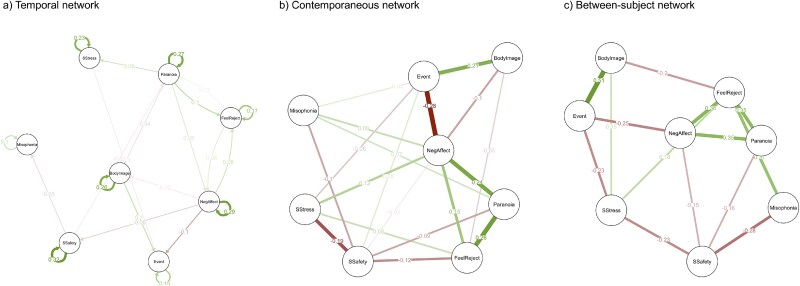
Temporal, Contemporaneous and Between-Subject Network Models Estimated for the Total Sample.

Figure 1A presents the estimated temporal network model for the total sample (*n* = 175). All 8 nodes showed a positive autocorrelation over time, with the strongest effects being observed for social safety, negative affect, and paranoid thoughts. A total of 14 nonzero edges were found (25% of the possible 56 edges, excluding autocorrelation edges), consisting of 7 positive and 7 negative edges. Paranoid thoughts significantly predicted negative affect, feelings of rejection, and negative body image over time and were significantly predicted only by feelings of rejection and social safety. A feedback loop mechanism was observed, with feelings of rejection being a significant predictor of paranoid thoughts, which in turn predicted further feelings of rejection. Among all the edges in the network, the strongest were paranoid thoughts predicting feelings of being rejected and negative affect predicting important/stressful events. The nodes with the highest in-expected influence (ie, those most strongly predicted by other nodes) were feeling rejected and negative affect. The nodes with the highest out-expected influence (ie, those most strongly predicting other nodes) were paranoid thoughts and negative affect.

Figure 3A presents the estimated temporal network model for the LP group (*n* = 103). Similarly, all 8 nodes showed a positive autocorrelation over time, with the strongest effects being observed for social safety, negative affect, and social stress. A total of 9 nonzero edges were found (16% of the possible 56 edges), consisting of 5 positive and 4 negative edges. Paranoid thoughts were significantly predicted by negative affect and misophonia symptoms, but paranoid thoughts themselves were not found to significantly predict other variables. Among all the edges in the network, the strongest were negative body image predicting important/stressful events and negative affect predicting important/stressful events. The nodes with the highest in-expected influence were feeling rejected and body image. The nodes with the highest out-expected influence were body image and negative affect.

Finally, Figure 3B presents the estimated temporal network model for the HP group (*n* = 72). All the 8 nodes showed a positive autocorrelation over time, with the strongest effects being observed for paranoid thoughts, social safety, and negative affect. A total of 13 nonzero edges were found (23% of the possible 56 edges), consisting of 9 positive and 4 negative edges. Paranoid thoughts significantly predicted negative affect, feelings of rejection, and social stress. In turn, paranoid thoughts were significantly predicted by feelings of rejection and social safety. Again, a feedback loop mechanism was observed, suggesting a bidirectional relationship between feelings of rejection and paranoid thoughts. Among all the edges in the network, the strongest were paranoid thoughts predicting feelings of being rejected and negative affect predicting social safety. The nodes with the highest in-expected influence were feelings of rejection and social stress. The nodes with the highest out-expected influence were paranoid thoughts and feelings of rejection.

The permutation tests revealed a significant difference between the LP and HP groups in 7 edges, including 2 autocorrelation and 5 temporal edges (see Figure 3C). The HP group showed a stronger autocorrelation effect for paranoid thoughts and stronger temporal effects for feelings of rejection predicting negative affect, feelings of rejection predicting social stress, and negative affect predicting social safety. Conversely, the LP group demonstrated a stronger autocorrelation effect for social stress and stronger temporal effect for important/stressful events predicting body image. The visual inspection indicated that the network model estimated for the HP group exhibited a greater number and stronger nonzero edges compared to the LP group. Additionally, the HP group temporal network model had no isolated nodes, in contrast to the LP group model, which showed an isolated node representing social safety.

**Figure 2:**
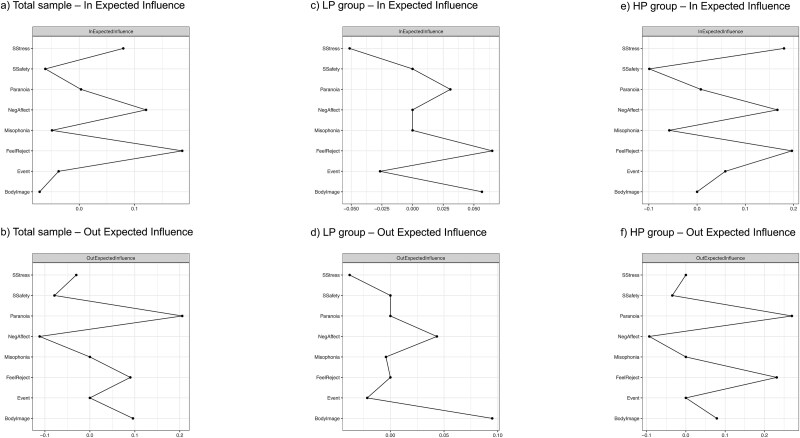
In- and Out-Expected Influence Values.

Table S2 (see online supplementary material) shows the edge values estimated for the temporal network models for the total sample, as well as for the LP and HP groups separately. In- and out-expected influence values for all nodes within the temporal network models are presented in Table S1 (see the online supplementary material), as well as in Figure 2A and B for the total sample, Figure 2C and D for the LP group, and Figure 2E and F for the HP group.

**Figure 3:**
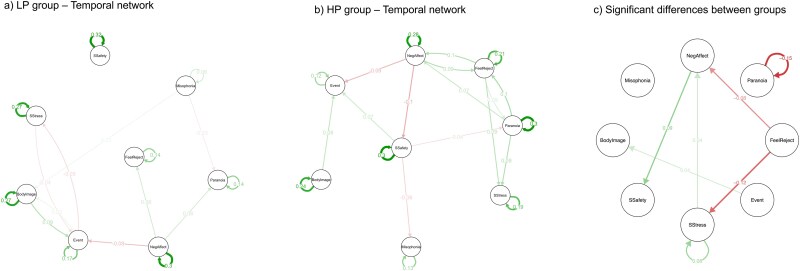
Temporal Network Models Estimated for the LP and HP Groups.

### Contemporaneous Network Estimation

The contemporaneous network models consist of 8 nodes (variables) and a total of 28 edges. Figure 1B presents the estimated contemporaneous network model for the total sample (*n* = 175). A total of 20 (71%) nonzero edges were identified, consisting of 10 positive and 10 negative edges. Paranoid thoughts turned out to be significantly and directly associated with 4 nodes—feelings of rejection, negative affect, social safety, and misophonia symptoms. Among those, the strongest edges represented links between paranoid thoughts and feelings of rejection and negative affect.

**Figure 4:**
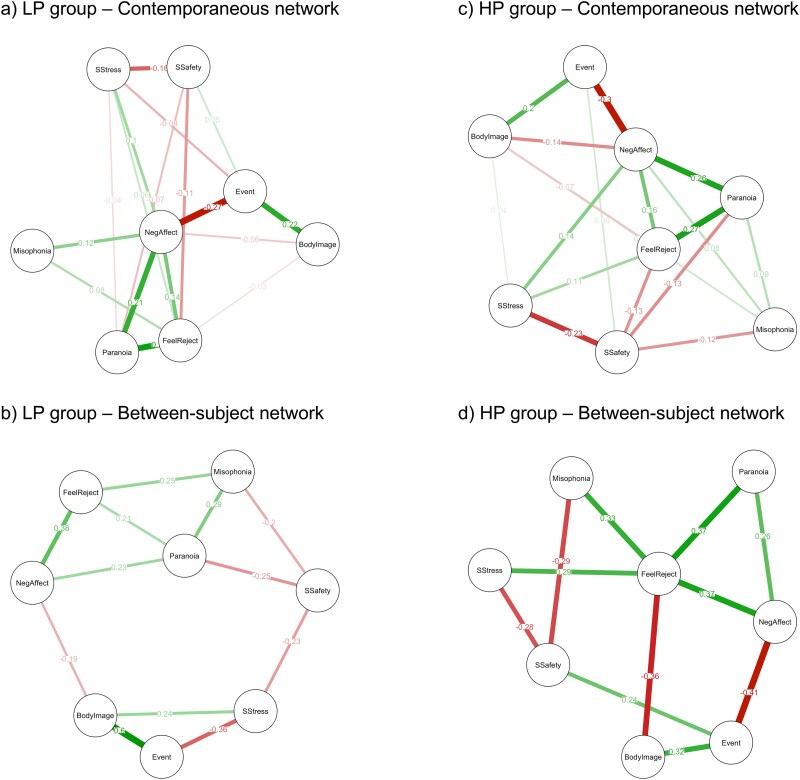
Contemporaneous and Between-Subject Network Models Estimated for the LP and HP Groups.

Figure 4A presents the estimated contemporaneous network model for the LP group (*n* = 103). A total of 17 (61%) nonzero edges were identified, consisting of 9 positive and 8 negative edges. Paranoid thoughts were significantly and directly associated with 4 nodes—feelings of rejection, negative affect, social stress, and social safety. Among those, the strongest edges represented links between paranoid thoughts and feelings of rejection and negative affect.

Figure 4C presents the estimated contemporaneous network model for the HP group (*n* = 72). A total of 18 (64%) nonzero edges were identified, consisting of 11 positive and 7 negative edges. Paranoid thoughts were significantly and directly associated with 4 nodes—feelings of rejection, negative affect, social safety, and misophonia symptoms. Among those, the strongest edges represented links between paranoid thoughts and feelings of rejection and negative affect.

The permutation tests revealed a significant difference between the LP and HP groups in 4 edges. The HP group showed a stronger association between negative affect and body image, and between social safety and misophonia symptoms, compared to the LP group. Conversely, the LP group demonstrated a stronger association between feelings of rejection and misophonia symptoms. Negative affect and feeling rejected seemed to act as mediators in the relationships between paranoid thoughts and the remaining variables in both groups.

Table S3 shows the edge values estimated for the contemporaneous network models for the total sample, as well as for the LP and HP groups separately.

### Between-Subject Network Estimation

The between-subject network models consist of 8 nodes (variables) and a total of 28 edges.

Figure 1C presents the estimated between-subject network model for the total sample (*n* = 175). A total of 14 (50%) nonzero edges were identified, consisting of 7 positive and 7 negative edges. Paranoid thoughts were significantly and directly associated with 3 nodes—negative affect, feelings of rejection, and social safety. Among those, the strongest edges represented links between paranoid thoughts and negative affect and feelings of rejection.

Figure 4B presents the estimated between-subject network model for the LP group (*n* = 103). A total of 12 (43%) nonzero edges were identified, consisting of 7 positive and 5 negative edges. Paranoid thoughts were found to be significantly and directly associated with 4 nodes—negative affect, feelings of rejection, social safety, and misophonia symptoms. Among those, the strongest edge represented a link between paranoid thoughts and misophonia symptoms.

Figure 4D presents the estimated between-subject network model for the HP group (*n* = 72). A total of 11 (39%) nonzero edges were identified, consisting of 7 positive and 4 negative edges. Paranoid thoughts were significantly and directly associated with 2 nodes—feelings of rejection and negative affect.

The permutation tests revealed a significant difference between the LP and HP groups in 3 edges. The HP group showed a stronger association between feelings of rejection and body image, and between important/stressful event and social safety, as compared to the LP group. Conversely, the LP group demonstrated a stronger association between important/stressful event and social stress.

Table S4 shows the edge values estimated for the between-subject network models for the total sample, as well as for the LP and HP groups separately.

## Discussion

This study employed ESM data and temporal network analysis to examine the dynamic interactions among factors contributing to a sense of vulnerability and their role in the development of paranoid thoughts.

The findings consistently indicate that social rejection, negative affect, and a perceived lack of social safety are central to paranoid thoughts. Among all the variables included in the network models, social rejection was the only one to exhibit a bidirectional relationship with paranoia. This suggests a two-way dynamic in which social rejection predicts an increase in paranoid thoughts, and conversely, paranoia predicts feelings of rejection. Whereas previous research has primarily focused on the unidirectional nature of this relationship,^11^^,^^13^ our findings highlight the importance of recognizing social rejection not only as a predictor but also as a consequence of paranoia. This self-perpetuating cycle can exacerbate both experiences, increasing emotional distress and potentially contributing to the onset of more severe symptoms. It has been observed that individuals with heightened rejection sensitivity tend to respond to rejection with withdrawal and isolation, thus further reinforcing the vicious cycle.^62^ Furthermore, feelings of social rejection and negative affect were not only the key components of temporal network models, but their associations with paranoid thoughts were also among the strongest relationships in both contemporaneous and between-subject networks, indicating both their simultaneous co-occurrence and a broader correlation, independent of individual differences. It is important to consider when interpreting these results that fears of social rejection and associated negative emotions constitute a fundamental component of paranoid thought content,^63^ which may contribute to a certain degree of overlap between these constructs. Nevertheless, it remains evident that social rejection and negative affect play a central role in paranoid thinking.

The primary goal of this study was to examine the interplay between various predictors of paranoid thoughts, hypothesized to contribute to their development through a shared effect on vulnerability. Somewhat unexpectedly, the estimated network models revealed that, although paranoia was predicted by some variables, it was not the most strongly *predicted* variable among those included in the model. Instead, it emerged as the strongest *predictor* of other variables, both in the total sample and the high-paranoia subgroup. Specifically, paranoid thoughts significantly predicted negative affect, social rejection, and social stress, while they were themselves directly predicted by social rejection and a lack of social safety. The bidirectional relationships between paranoid thoughts and their psychosocial correlates have been demonstrated in previous studies.^25^^,^^64–66^ Indeed, paranoid thoughts have been shown to arise from the interaction of multiple risk factors, including worry, sleep disturbances, stress, and depressive symptoms. However, they can also further intensify stress, worry, negative body image, and impair social functioning. Notably, alongside negative affect and a reduced sense of social safety, paranoid thoughts demonstrated the strongest autocorrelation over time, suggesting a degree of persistence—individuals experiencing high levels of paranoia at one point are likely to continue experiencing paranoia in the future, potentially exacerbating other symptoms. On the other hand, the fact that paranoid thoughts were not directly predicted by many proposed variables in the estimated network models does not necessarily imply an absence of causal relationships. Instead, these associations may be mediated by other factors, such as social rejection or negative emotions, which exhibited the highest in-expected influence, meaning they were the variables most strongly predicted by other variables in the network. Social rejection emerged as a direct predictor of paranoid thoughts. Aligning with the approach adopted in this study, it may be suggested that stressful events, a negative self-view, or negative emotional states (eg, misophonic reactions) may first impact rejection sensitivity and feelings of inferiority, which in turn foster paranoid thoughts. In contrast, in the control group, paranoia did not significantly predict any other variable in the network. Instead, negative emotions and negative body image had the strongest predictive effect. Since paranoia was not central to this group’s psychological dynamics, other vulnerability-related factors, such as body image concerns, played a more prominent role. Over time, consistent with the network approach to psychopathology,^31^^,^^32^ these factors may contribute to worsening symptoms and activate interconnected psychological processes, potentially leading to more severe consequences, such as the development of paranoid thoughts. However, it is important to acknowledge that the LP group was characterized by a very low baseline level of paranoid thoughts, which was also reflected in the ESM measures. Consequently, these findings are likely attributable, in large part, to floor effects and the minimal variance in paranoia over time.

Although the results revealed several significant differences between the groups, they were relatively few and did not predominantly involve paranoid thoughts. However, a visual inspection of the estimated models indicated a greater number of significant and stronger associations between variables in the high-paranoia group. This suggests that as paranoid thoughts increase, it is not necessarily the structure of the network that changes, but rather the strength of the relationships between factors (ie, network connectivity). These findings may be cautiously interpreted in the context of the complex dynamic systems theory,^67^ which suggests that gradual changes within a system may eventually reach a critical threshold, triggering a sudden shift to more severe psychological states. On the other hand, symptom deactivation—such as through psychological interventions—may have a cascading beneficial effect. For instance, previous research revealed that targeting worry^68^ and insomnia^69^ significantly reduced paranoid thoughts. Therefore, recognizing early warning signals that destabilize the system could facilitate timely interventions that specifically address the most central symptoms, potentially preventing them from developing into full-blown disorders.

When interpreting the results of this study, it is important to consider its limitations. First, although the factors selected as predictors of a sense of vulnerability in the context of paranoid thoughts were based on mechanisms described in the existing literature,^70^ it remains unclear whether they fully capture the underlying processes. For instance, the model did not account for global self-esteem, which often represents the concerns about social evaluation. Importantly, the variables included in the model were intended to represent broader psychological constructs contributing to interpersonal vulnerability—such as fear of rejection, negative self-views, and perceptions of others and the world as threatening—rather than isolated factors. It is plausible that alternative variables could similarly capture the mechanisms described. For instance, misophonia was introduced as a novel element to reflect the perception of social threat. However, given the early stage of research on the link between misophonia and paranoia, further studies are needed to fully understand its role and implications. Additionally, while this study focused specifically on factors that may increase a sense of vulnerability, incorporating other potential triggers of paranoid thoughts, such as sleep disturbances and substance use, could provide further insights as the inclusion of additional factors might alter the network’s dynamics. Future research should therefore examine vulnerability-related factors alongside a broader range of correlates of paranoid thoughts^9^ to determine whether the same variables remain central. Moreover, future studies could include additional groups along the paranoia continuum, such as individuals at ultrahigh risk or patients with schizophrenia experiencing persecutory delusions. This would allow for the investigation of whether the structure of paranoid thoughts remains consistent while the strength of relationships between variables further intensifies in clinical populations. It is also possible that the absence of structural differences observed in this study resulted from the groups being too similar to detect meaningful distinctions. Notably, relative to the LP group, a significantly higher proportion of HP participants had a diagnosis of, or met the MINI criteria for, comorbid disorders (eg, depression). Therefore, the observed between-group differences should be interpreted with caution, as they may also be attributable to the effects of comorbidity, including its consequences, such as stigma, rather than paranoid thoughts per se. Another limitation is that some variables were measured using only a single item. In particular, the measures of misophonia symptoms and body image were employed for the first time in the ESM study protocols, underscoring the need for their validation in other samples. Furthermore, social isolation (and thus social stress and social safety) was assessed solely on the basis of the physical presence of others, without taking into account any remote or virtual social interactions, limiting our understanding of the full spectrum of participants’ social experiences. Lastly, fluctuations in variables over time do not necessarily imply causality. Thus, integrating intensive longitudinal studies with experimental research is essential for examining causal relationships.

To conclude, this study is the first to use intensive longitudinal data to build a complex temporal network model of the mechanisms behind paranoia. The results identify social rejection and negative affect as the most central symptoms, each strongly influencing and being influenced by other variables, and underscore the bidirectional relationship between paranoia and social rejection. Although the overall network structure remains stable, higher levels of paranoia intensify symptom interconnectedness, highlighting the need for early intervention.

## Supplementary Material

Supplementary_materials_sgaf021

## Data Availability

The data that support the findings of this study are available from the corresponding author [PB] upon reasonable request.
