# Supplementary material for: “Rejection Makes Me Suspicious”: Complex Temporal Network Approach to the Dynamics of Real-Time Paranoid Thoughts and Psychological Vulnerability
Source: Schizophr Bull Open. 2025 Sep 25;6(1):sgaf021. doi: 10.1093/schizbullopen/sgaf021 (PMC12551459; doi:10.1093/schizbullopen/sgaf021)
Supplement: Supplementary_materials_sgaf021 [file supplementary_materials_sgaf021.pdf]

## Supplementary Materials

### ‘Rejection makes me suspicious’: Complex temporal network approach to the dynamics of real-time paranoid thoughts and psychological vulnerability

**Supplementary Table 1.** In- and out-expected influence values.

| Node | Total sample | | LP | | HP | |
|---|---|---|---|---|---|---|
| | *In-expected influence* | *Out-expected influence* | *In-expected influence* | *Out-expected influence* | *In-expected influence* | *Out-expected influence* |
| **Negative affect** | 0.121 | -0.112 | 0.00 | 0.043 | 0.166 | -0.093 |
| **Paranoia** | 0.003 | 0.205 | 0.031 | 0.00 | 0.008 | 0.270 |
| **Social rejection** | 0.186 | 0.09 | 0.065 | 0.00 | 0.196 | 0.231 |
| **Event** | -0.037 | 0.00 | -0.026 | -0.022 | 0.058 | 0.00 |
| **Social stress** | 0.079 | -0.03 | -0.051 | -0.038 | 0.180 | 0.00 |
| **Social safety** | -0.061 | -0.079 | 0.00 | 0.00 | -0.10 | -0.035 |
| **Body image** | -0.071 | 0.096 | 0.057 | 0.095 | 0.00 | 0.079 |
| **Misophonia** | -0.049 | 0.00 | 0.00 | -0.004 | -0.058 | 0.00 |

**Supplementary Table 2.** Edge weights in the temporal network models and path differences between groups.

| Path | | Total sample | LP | HP | Difference *(LP-HP)* | *p* |
|---|---|---|---|---|---|---|
| **From** $_{(t-1)}$ | **To** $_{(t)}$ | | | | | |
| NegAffect | NegAffect | **0.29** | **0.30** | **0.28** | 0.02 | 0.633 |
| NegAffect | Paranoia | 0.02 | **0.06** | 0.01 | 0.05 | 0.261 |
| NegAffect | FeelReject | **0.08** | **0.07** | **0.09** | -0.03 | 0.513 |
| NegAffect | Event | **-0.10** | **-0.08** | **-0.09** | 0.01 | 0.909 |
| NegAffect | SStress | 0.04 | 0.02 | 0.04 | -0.02 | 0.673 |
| NegAffect | SSafety | **-0.06** | -0.01 | **-0.10** | **0.09** | **0.045** |
| NegAffect | BodyImage | **-0.03** | -0.02 | -0.04 | 0.03 | 0.385 |
| NegAffect | Misophonia | -0.01 | -0.01 | -0.01 | 0.00 | 0.988 |
| Paranoia | NegAffect | **0.06** | 0.04 | **0.07** | -0.04 | 0.4 |
| Paranoia | Paranoia | **0.27** | **0.14** | **0.30** | **-0.15** | **0.021** |
| Paranoia | FeelReject | **0.10** | 0.03 | **0.10** | -0.08 | 0.225 |
| Paranoia | Event | -0.02 | 0.01 | -0.04 | 0.06 | 0.401 |
| Paranoia | SStress | **0.08** | 0.03 | **0.10** | -0.07 | 0.248 |
| Paranoia | SSafety | -0.03 | -0.02 | -0.04 | 0.01 | 0.784 |
| Paranoia | BodyImage | **-0.04** | -0.01 | -0.06 | 0.05 | 0.336 |
| Paranoia | Misophonia | 0.06 | 0.03 | 0.06 | -0.03 | 0.65 |
| FeelReject | NegAffect | **0.06** | 0.01 | **0.10** | **-0.08** | **0.025** |
| FeelReject | Paranoia | **0.03** | 0.02 | **0.05** | -0.03 | 0.357 |
| FeelReject | FeelReject | **0.17** | **0.14** | **0.21** | -0.07 | 0.166 |
| FeelReject | Event | -0.01 | 0.02 | -0.04 | 0.05 | 0.283 |
| FeelReject | SStress | 0.03 | -0.03 | **0.09** | **-0.12** | **0.003** |
| FeelReject | SSafety | 0.01 | 0.02 | -0.01 | 0.02 | 0.557 |
| FeelReject | BodyImage | -0.01 | -0.02 | 0.01 | -0.03 | 0.294 |
| FeelReject | Misophonia | 0.03 | 0.00 | 0.05 | -0.05 | 0.303 |
| Event | NegAffect | -0.01 | 0.00 | -0.01 | 0.01 | 0.689 |
| Event | Paranoia | 0.00 | 0.02 | 0.00 | 0.02 | 0.279 |
| Event | FeelReject | 0.01 | 0.00 | 0.01 | -0.01 | 0.658 |
| Event | Event | **0.15** | **0.17** | **0.12** | 0.05 | 0.172 |
| Event | SStress | **-0.03** | **-0.05** | 0.00 | -0.05 | 0.111 |
| Event | SSafety | -0.01 | 0.01 | -0.02 | 0.03 | 0.148 |
| Event | BodyImage | 0.01 | **0.03** | -0.01 | **0.04** | **0.033** |
| Event | Misophonia | 0.01 | -0.01 | 0.02 | -0.04 | 0.315 |
| SStress | NegAffect | 0.00 | 0.02 | -0.02 | **0.04** | **0.026** |
| SStress | Paranoia | 0.00 | -0.01 | 0.00 | -0.01 | 0.535 |
| SStress | FeelReject | 0.00 | 0.01 | -0.01 | 0.01 | 0.563 |
| SStress | Event | **-0.03** | **-0.04** | -0.01 | -0.02 | 0.337 |

| | | | | | | |
|---|---|---|---|---|---|---|
| SStress | SStress | **0.23** | **0.27** | **0.19** | **0.08** | **0.035** |
| SStress | SSafety | 0.01 | 0.01 | 0.01 | 0.00 | 0.923 |
| SStress | BodyImage | 0.00 | -0.01 | 0.01 | -0.02 | 0.26 |
| SStress | Misophonia | -0.01 | 0.00 | -0.02 | 0.02 | 0.449 |
| SSafety | NegAffect | -0.01 | 0.01 | -0.02 | 0.03 | 0.224 |
| SSafety | Paranoia | **-0.03** | -0.02 | **-0.04** | 0.02 | 0.416 |
| SSafety | FeelReject | -0.02 | -0.01 | -0.03 | 0.02 | 0.551 |
| SSafety | Event | 0.03 | 0.00 | **0.07** | -0.07 | 0.078 |
| SSafety | SStress | -0.01 | 0.00 | -0.03 | 0.02 | 0.621 |
| SSafety | SSafety | **0.32** | **0.32** | **0.30** | 0.03 | 0.501 |
| SSafety | BodyImage | 0.01 | -0.01 | 0.03 | -0.05 | 0.055 |
| SSafety | Misophonia | **-0.05** | -0.04 | **-0.06** | 0.02 | 0.598 |
| BodyImage | NegAffect | **-0.03** | -0.03 | -0.03 | 0.01 | 0.849 |
| BodyImage | Paranoia | -0.02 | -0.03 | -0.03 | -0.01 | 0.802 |
| BodyImage | FeelReject | -0.01 | -0.02 | 0.00 | -0.02 | 0.507 |
| BodyImage | Event | **0.10** | **0.10** | **0.08** | 0.01 | 0.793 |
| BodyImage | SStress | 0.00 | 0.01 | 0.01 | 0.00 | 0.996 |
| BodyImage | SSafety | 0.03 | -0.01 | 0.04 | -0.05 | 0.125 |
| BodyImage | BodyImage | **0.26** | **0.27** | **0.24** | 0.03 | 0.47 |
| BodyImage | Misophonia | -0.02 | -0.03 | 0.00 | -0.03 | 0.407 |
| Misophonia | NegAffect | 0.00 | -0.01 | 0.01 | -0.02 | 0.513 |
| Misophonia | Paranoia | -0.01 | **-0.03** | 0.00 | -0.03 | 0.324 |
| Misophonia | FeelReject | -0.01 | -0.02 | 0.00 | -0.02 | 0.525 |
| Misophonia | Event | 0.01 | -0.01 | 0.03 | -0.04 | 0.304 |
| Misophonia | SStress | 0.01 | 0.02 | 0.00 | 0.02 | 0.55 |
| Misophonia | SSafety | 0.00 | -0.03 | 0.01 | -0.04 | 0.268 |
| Misophonia | BodyImage | 0.02 | **0.03** | 0.02 | 0.00 | 0.883 |
| Misophonia | Misophonia | **0.10** | **0.06** | **0.13** | -0.06 | 0.122 |

**Supplementary Table 3.** Edge weights in the contemporaneous network models and path differences between groups.

| **Path** | | **Total sample** | **LP** | **HP** | **Difference** *(LP-HP)* | ***p*** |
|---|---|---|---|---|---|---|
| NegAffect | Paranoia | **0.24** | **0.21** | **0.26** | 0.05 | 0.21 |
| NegAffect | FeelReject | **0.15** | **0.14** | **0.16** | -0.03 | 0.537 |
| NegAffect | Event | **-0.28** | **-0.27** | **-0.30** | 0.05 | 0.238 |
| NegAffect | SStress | **0.12** | **0.10** | **0.14** | -0.05 | 0.161 |
| NegAffect | SSafety | **-0.03** | -0.02 | -0.04 | 0.01 | 0.8 |
| NegAffect | BodyImage | **-0.1** | **-0.06** | **-0.14** | **0.07** | **0.046** |
| NegAffect | Misophonia | **0.09** | **0.12** | **0.08** | 0.04 | 0.325 |
| Paranoia | FeelReject | **0.26** | **0.25** | **0.27** | -0.01 | 0.826 |
| Paranoia | Event | -0.01 | -0.01 | -0.02 | 0.01 | 0.868 |
| Paranoia | SStress | -0.01 | -0.04 | 0.01 | -0.05 | 0.157 |
| Paranoia | SSafety | **-0.09** | **-0.07** | **-0.13** | 0.08 | 0.066 |
| Paranoia | BodyImage | 0.02 | 0.01 | 0.02 | -0.01 | 0.751 |
| Paranoia | Misophonia | **0.07** | 0.02 | **0.09** | -0.07 | 0.108 |
| FeelReject | Event | -0.01 | 0.00 | 0.00 | 0.00 | 0.943 |
| FeelReject | SStress | **0.09** | **0.06** | **0.11** | -0.06 | 0.145 |
| FeelReject | SSafety | **-0.12** | **-0.11** | **-0.13** | 0.03 | 0.48 |
| FeelReject | BodyImage | **-0.05** | **-0.03** | **-0.07** | 0.03 | 0.365 |
| FeelReject | Misophonia | 0.02 | **0.08** | 0.00 | **0.09** | **0.033** |
| Event | SStress | **-0.06** | **-0.08** | -0.04 | -0.04 | 0.222 |
| Event | SSafety | **0.05** | **0.06** | **0.06** | 0.00 | 0.958 |
| Event | BodyImage | **0.21** | **0.22** | **0.20** | 0.03 | 0.47 |
| Event | Misophonia | -0.01 | -0.02 | -0.01 | -0.01 | 0.849 |
| SStress | SSafety | **-0.20** | **-0.16** | **-0.23** | 0.07 | 0.08 |
| SStress | BodyImage | 0.01 | -0.03 | **0.04** | **-0.07** | **0.022** |
| SStress | Misophonia | 0.03 | 0.03 | 0.02 | 0.01 | 0.821 |
| SSafety | BodyImage | 0.02 | 0.01 | 0.03 | -0.03 | 0.446 |
| SSafety | Misophonia | **-0.10** | -0.05 | **-0.12** | **0.08** | **0.026** |
| BodyImage | Misophonia | **0.05** | 0.02 | **0.06** | -0.04 | 0.286 |

**Supplementary Table 4.** Edge weights in the between-subject network models and path differences between groups.

| Path | | Total sample | LP | HP | Difference *(LP-HP)* | *p* |
|---|---|---|---|---|---|---|
| NegAffect | Paranoia | **0.35** | **0.23** | **0.26** | -0.04 | 0.867 |
| NegAffect | FeelReject | **0.36** | **0.38** | **0.37** | 0.01 | 0.966 |
| NegAffect | Event | **-0.25** | -0.06 | **-0.41** | 0.37 | 0.149 |
| NegAffect | SStress | 0.08 | 0.15 | 0.10 | 0.06 | 0.766 |
| NegAffect | SSafety | **-0.15** | 0.00 | -0.12 | 0.12 | 0.565 |
| NegAffect | BodyImage | 0.02 | **-0.19** | 0.20 | -0.39 | 0.052 |
| NegAffect | Misophonia | 0.06 | -0.01 | 0.03 | -0.05 | 0.798 |
| Paranoia | FeelReject | **0.35** | **0.21** | **0.37** | -0.19 | 0.429 |
| Paranoia | Event | -0.03 | 0.08 | -0.13 | 0.23 | 0.105 |
| Paranoia | SStress | -0.12 | 0.05 | -0.19 | 0.25 | 0.15 |
| Paranoia | SSafety | **-0.16** | **-0.25** | -0.09 | -0.18 | 0.357 |
| Paranoia | BodyImage | 0.03 | 0.10 | 0.07 | 0.04 | 0.8 |
| Paranoia | Misophonia | -0.02 | **0.30** | -0.06 | 0.34 | 0.141 |
| FeelReject | Event | 0.09 | -0.17 | 0.14 | -0.30 | 0.084 |
| FeelReject | SStress | **0.18** | -0.01 | **0.29** | -0.29 | 0.082 |
| FeelReject | SSafety | -0.03 | -0.03 | 0.04 | -0.06 | 0.819 |
| FeelReject | BodyImage | **-0.20** | 0.08 | **-0.36** | **0.47** | **0.016** |
| FeelReject | Misophonia | **0.31** | **0.25** | **0.33** | -0.08 | 0.753 |
| Event | SStress | **-0.23** | **-0.36** | 0.00 | **-0.36** | **0.03** |
| Event | SSafety | 0.03 | -0.07 | **0.24** | **-0.32** | **0.043** |
| Event | BodyImage | **0.51** | **0.60** | **0.32** | 0.26 | 0.082 |
| Event | Misophonia | 0.06 | -0.01 | 0.13 | -0.16 | 0.362 |
| SStress | SSafety | **-0.23** | **-0.23** | **-0.28** | 0.03 | 0.837 |
| SStress | BodyImage | **0.15** | **0.24** | 0.10 | 0.16 | 0.384 |
| SStress | Misophonia | -0.03 | -0.07 | -0.07 | -0.01 | 0.971 |
| SSafety | BodyImage | 0.10 | 0.05 | 0.21 | -0.16 | 0.323 |
| SSafety | Misophonia | **-0.28** | **-0.20** | **-0.30** | 0.10 | 0.595 |
| BodyImage | Misophonia | -0.01 | -0.12 | 0.12 | -0.25 | 0.155 |
